# The genome sequence of the straw spear-moss, *Straminergon stramineum* (Dicks. ex Brid.) Hedenas, 1993

**DOI:** 10.12688/wellcomeopenres.23432.1

**Published:** 2024-12-03

**Authors:** David Bell, Liz Kungu, David Long, Royal Botanic Garden Edinburgh Genome Acquisition Lab, Plant Genome Sizing collective, Darwin Tree of Life Barcoding collective, Wellcome Sanger Institute Tree of Life Management, Samples and Laboratory team, Wellcome Sanger Institute Scientific Operations: Sequencing Operations, Wellcome Sanger Institute Tree of Life Core Informatics team, Tree of Life Core Informatics collective, Darwin Tree of Life Consortium

**Affiliations:** 1 Royal Botanic Garden Edinburgh, Edinburgh, Scotland, UK

**Keywords:** Straminergon stramineum, straw spear-moss, genome sequence, chromosomal, Hypnales

## Abstract

We present a genome assembly from a haploid *Straminergon stramineum* gametophyte (the straw spear-moss; Streptophyta; Bryopsida; Hypnales; Calliergonaceae). The genome sequence spans 326.30 megabases. Most of the assembly is scaffolded into 11 chromosomal pseudomolecules. The mitochondrial and plastid genome assemblies have lengths of 104.6 kilobases and 124.69 kilobases, respectively.

## Species taxonomy

Eukaryota; Viridiplantae; Streptophyta; Streptophytina; Embryophyta; Bryophyta; Bryophytina; Bryopsida; Bryidae; Hypnanae; Hypnales; Calliergonaceae; *Straminergon*; *Straminergon stramineum* (Dicks. ex Brid.) Hedenas, 1993 (NCBI:txid249548).

## Background

*Straminergon stramineum* (Dicks. ex Brid.) Hedenäs, also known as straw spear-moss, is a pleurocarpous moss widely distributed in the northern hemisphere, and also known from northern South America, Australia, and New Zealand. It is a common species of wet, peaty ground across much of Britain and Ireland, often growing as scattered shoots among other bryophytes in flushes and bogs ( Blockeel *et al.*, 2014).

Hedenäs (1993) established the genus *Straminergon* to accommodate the sole species *S. stramineum*, which had previously been placed in the genus *Calliergon*.

*Straminergon stramineum* is a dioicous species, producing male and female reproductive structures on separate plants. Capsules are rarely produced in Britain and Ireland.

The genome of *Straminergon stramineum* was sequenced as part of the Darwin Tree of Life Project, a collaborative effort to sequence all named eukaryotic species in Britain and Ireland. The chromosomally complete genome presented here is consistent with chromosome counts reported from England, Poland and Japan ( Fritsch, 1991) and genome size estimates reported by Voglmayr (2000). We anticipate this high-quality reference genome will be a valuable genomic resource for a range of future studies.

## Genome sequence report

**Figure 1.**
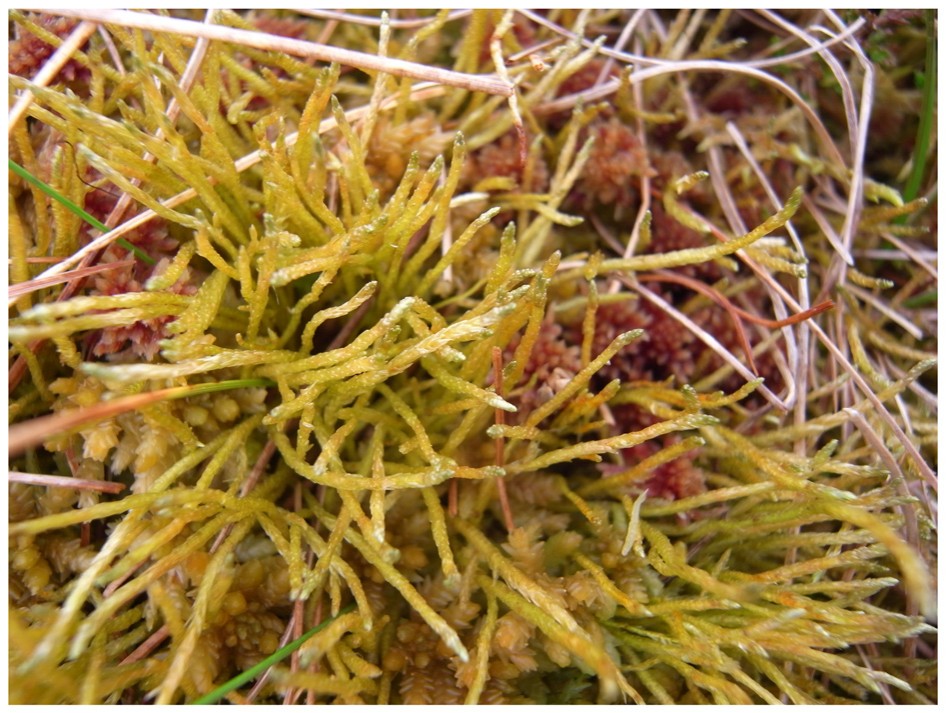
Photograph of the *Straminergon stramineum* population from which samples used for genome sequencing were taken.

The genome of a *Straminergon stramineum* gametophyte ( Figure 1) was sequenced using Pacific Biosciences single-molecule HiFi long reads, generating a total of 19.66 Gb (gigabases) from 2.00 million reads, providing approximately 83-fold coverage. Using flow cytometry, the genome size (1C-value) was estimated to be 0.41 pg, equivalent to 400 Mb. Primary assembly contigs were scaffolded with chromosome conformation Hi-C data, which produced 103.49 Gb from 685.35 million reads. Specimen and sequencing details are provided in Table 1.

**Table 1.** Specimen and sequencing data for *Straminergon stramineum*.

Project information			
**Study title**	Straminergon stramineum (straw spear-moss)		
**Umbrella BioProject**	PRJEB70978		
**Species**	*Straminergon stramineum*		
**BioSample**	SAMEA10904354		
**NCBI taxonomy ID**	249548		
Specimen information			
**Technology**	**ToLID**	**BioSample accession**	**Organism part**
**PacBio long read sequencing**	cbStrStra4	SAMEA10904554	shoot
**Hi-C sequencing**	cbStrStra1	SAMEA10904551	shoot
Sequencing information			
**Platform**	**Run accession**	**Read count**	**Base count (Gb)**
**Hi-C Illumina NovaSeq 6000**	ERR12356315	6.85e+08	103.49
**PacBio Sequel IIe**	ERR12370310	2.00e+06	19.66

**Figure 2.**
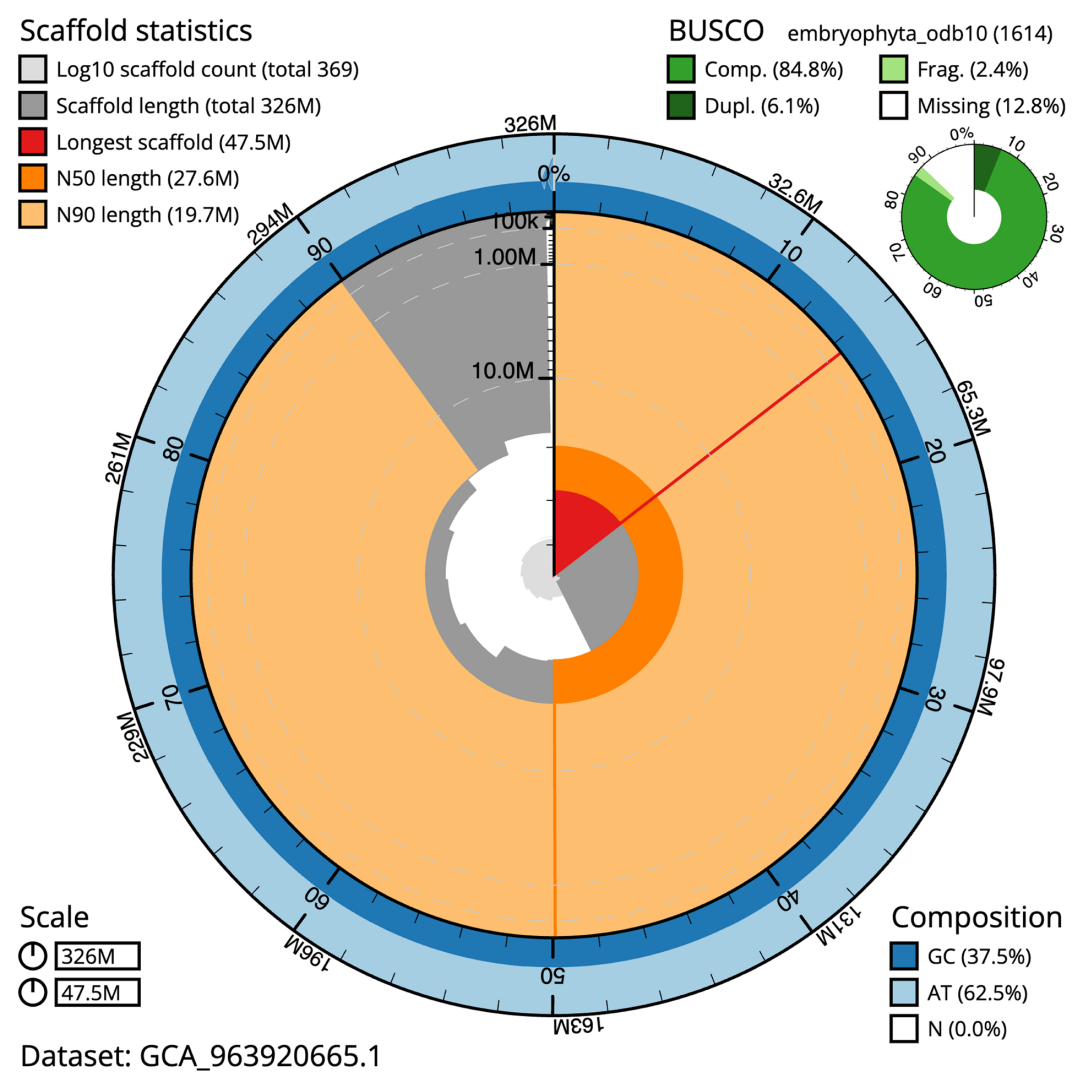
Genome assembly of *Straminergon stramineum*, cbStrStra4.1: metrics. The BlobToolKit snail plot shows N50 metrics and BUSCO gene completeness. The main plot is divided into 1,000 bins around the circumference with each bin representing 0.1% of the 326,482,466 bp assembly. The distribution of scaffold lengths is shown in dark grey with the plot radius scaled to the longest scaffold present in the assembly (47,471,227 bp, shown in red). Orange and pale-orange arcs show the N50 and N90 scaffold lengths (27,619,532 and 19,715,216 bp), respectively. The pale grey spiral shows the cumulative scaffold count on a log scale with white scale lines showing successive orders of magnitude. The blue and pale-blue area around the outside of the plot shows the distribution of GC, AT and N percentages in the same bins as the inner plot. A summary of complete, fragmented, duplicated and missing BUSCO genes in the embryophyta_odb10 set is shown in the top right. An interactive version of this figure is available at https://blobtoolkit.genomehubs.org/view/Straminergon_stramineum/dataset/GCA_963920665.1/snail.

**Figure 3.**
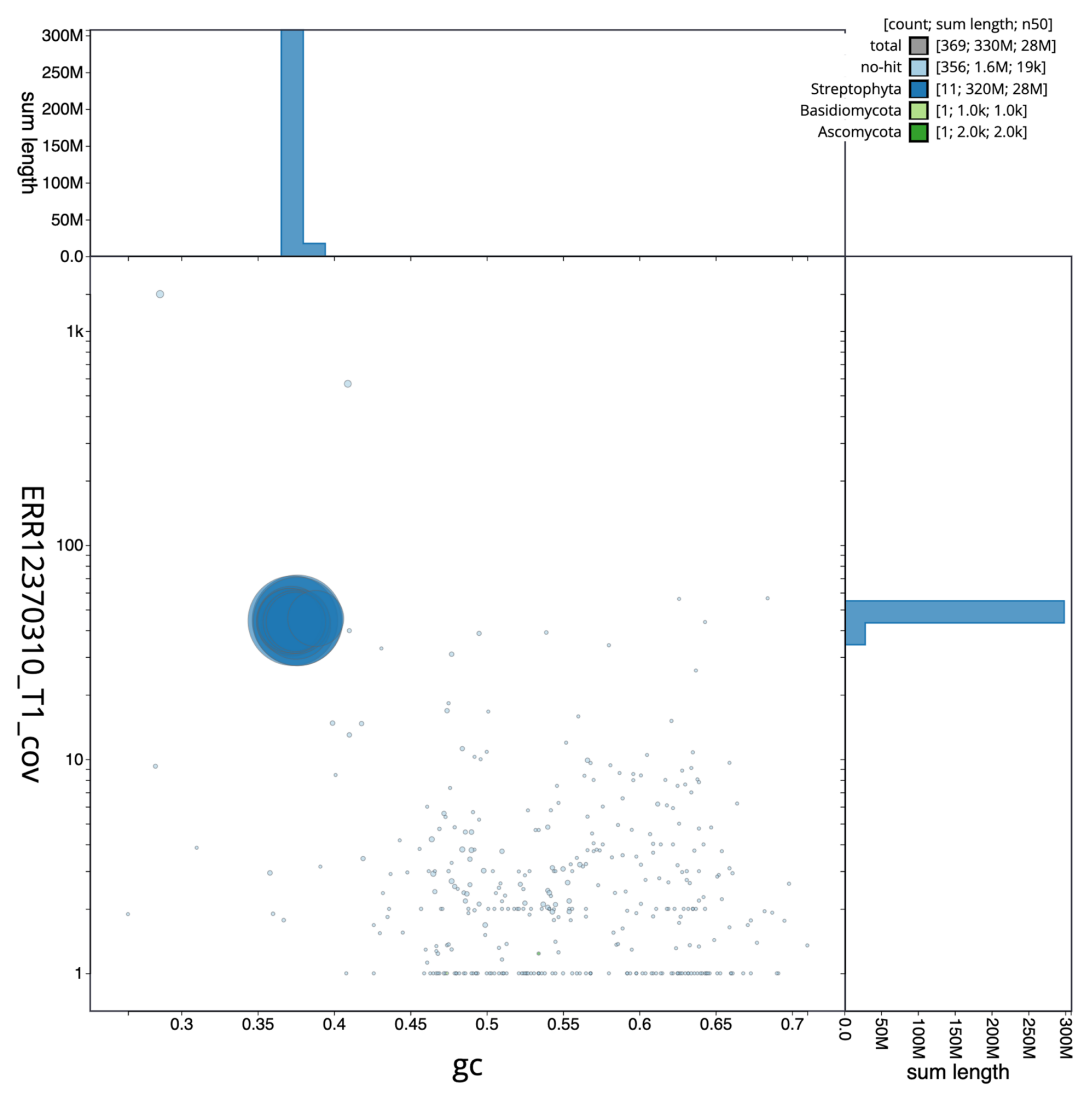
Genome assembly of *Straminergon stramineum*, cbStrStra4.1: BlobToolKit GC-coverage plot. Scaffolds are coloured by phylum. Circles are sized in proportion to scaffold length. Histograms show the distribution of scaffold length sum along each axis. An interactive version of this figure is available at https://blobtoolkit.genomehubs.org/view/Straminergon_stramineum/dataset/GCA_963920665.1/blob.

**Figure 4.**
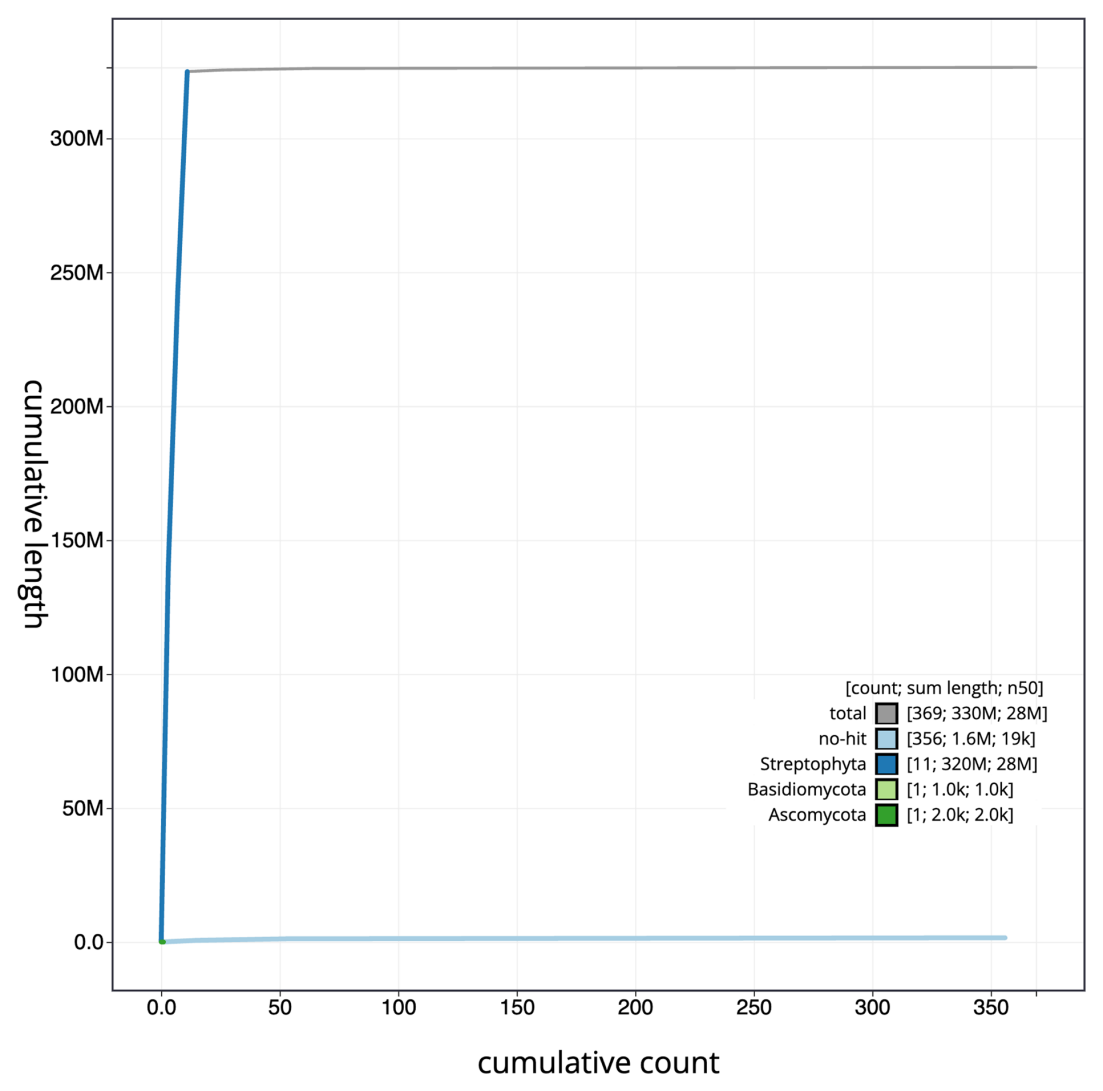
Genome assembly of *Straminergon stramineum*, cbStrStra4.1: BlobToolKit cumulative sequence plot. The grey line shows cumulative length for all scaffolds. Coloured lines show cumulative lengths of scaffolds assigned to each phylum using the buscogenes taxrule. An interactive version of this figure is available at https://blobtoolkit.genomehubs.org/view/Straminergon_stramineum/dataset/GCA_963920665.1/cumulative.

**Figure 5.**
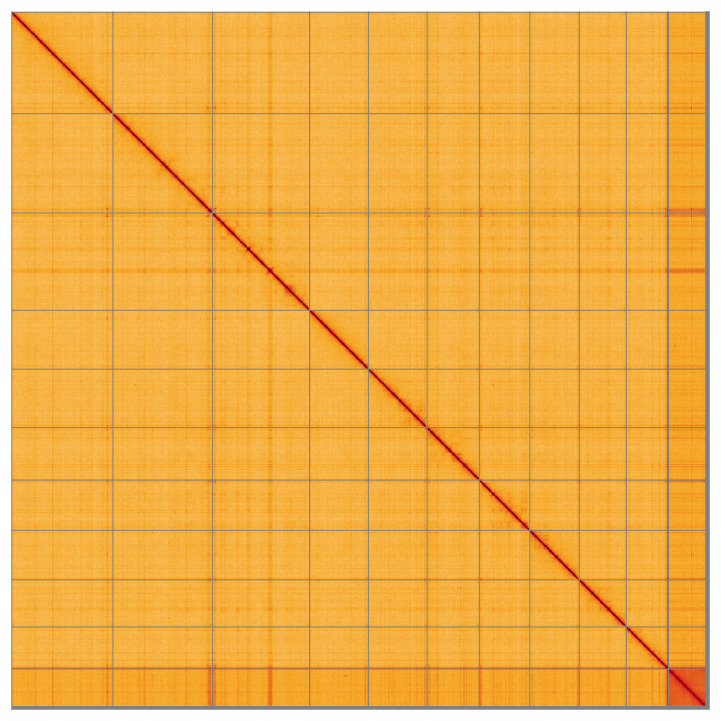
Genome assembly of *Straminergon stramineum*, cbStrStra4.1: Hi-C contact map of the cbStrStra4.1 assembly, visualised using HiGlass. Chromosomes are shown in order of size from left to right and top to bottom. An interactive version of this figure may be viewed at https://genome-note-higlass.tol.sanger.ac.uk/l/?d=eioniLUdQKGONAQCEtHJyw.

Manual assembly curation corrected 118 missing joins or mis-joins, reducing the assembly length by 1.91%, and increasing the scaffold N50 by 2.85%. The final assembly has a total length of 326.30 Mb in 367 sequence scaffolds with a scaffold N50 of 27.6 Mb ( Table 2), with a total of 515 gaps. The snail plot in Figure 2 provides a summary of the assembly statistics, while the distribution of assembly scaffolds on GC proportion and coverage is shown in Figure 3. The cumulative assembly plot in Figure 4 shows curves for subsets of scaffolds assigned to different phyla. Most (99.51%) of the assembly sequence was assigned to 11 chromosomal-level scaffolds. Chromosome-scale scaffolds confirmed by the Hi-C data are named in order of size ( Figure 5; Table 3). While not fully phased, the assembly deposited is of one haplotype. Contigs corresponding to the second haplotype have also been deposited. The mitochondrial and plastid genomes were also assembled and can be found as contigs within the multifasta file of the genome submission.

**Table 2.** Genome assembly data for *Straminergon stramineum*, cbStrStra4.1.

Genome assembly		
Assembly name	cbStrStra4.1	
Assembly accession	GCA_963920665.1	
Span (Mb)	326.30	
Number of contigs	884	
Number of scaffolds	367	
Longest scaffold (Mb)	47.47	
Assembly metrics *		*Benchmark*
Contig N50 length (Mb)	1.1	*≥ 1 Mb*
Scaffold N50 length (Mb)	27.6	*= chromosome N50*
Consensus quality (QV)	62.9	*≥ 40*
*k*-mer completeness	100.0%	*≥ 95%*
BUSCO **	C:84.4%[S:78.3%,D:6.1%],F:2.9%,M:12.7%,n:1,614	*S > 90%, D < 5%*
Percentage of assembly mapped to chromosomes	99.51%	*≥ 90%*
Sex chromosomes	None	*localised homologous* *pairs*
Organelles	Mitochondrial genome: 104.6 kb; Plastid genome: 124.69 kb	*complete single* *alleles*

* Assembly metric benchmarks are adapted from column VGP-2020 of “Table 1: Proposed standards and metrics for defining genome assembly quality” from Rhie *et al.* (2021).

** BUSCO scores based on the embryophyta_odb10 BUSCO set using version 5.4.3. C = complete [S = single copy, D = duplicated], F = fragmented, M = missing, n = number of orthologues in comparison. A full set of BUSCO scores is available at https://blobtoolkit.genomehubs.org/view/Straminergon_stramineum/dataset/GCA_963920665.1/busco.

**Table 3.** Chromosomal pseudomolecules in the genome assembly of Straminergon stramineum, cbStrStra4.

INSDC accession	Name	Length (Mb)	GC%
OY987263.1	1	47.47	37.5
OY987264.1	2	46.5	37.5
OY987265.1	3	45.43	37.5
OY987266.1	4	27.62	37.0
OY987267.1	5	27.38	37.5
OY987268.1	6	24.56	37.0
OY987269.1	7	23.57	37.5
OY987270.1	8	23.16	37.0
OY987271.1	9	22.04	37.5
OY987272.1	10	19.72	37.5
OY987273.1	11	17.45	39.0
OY987274.1	MT	0.1	41.0
OY987275.1	Pltd	0.12	28.5

The estimated Quality Value (QV) of the final assembly is 62.9 with *k*-mer completeness of 100.0%, and the assembly has a BUSCO v completeness of 84.4% (single = 78.3%, duplicated = 6.1%), using the embryophyta_odb10 reference set ( *n* = 1,614).

Metadata for specimens, BOLD barcode results, spectra estimates, sequencing runs, contaminants and pre-curation assembly statistics are given at https://links.tol.sanger.ac.uk/species/249548.

## Methods

### Sample acquisition, DNA barcoding and genome size estimation

Specimens of *Straminergon stramineum* were collected from Brad Moss north of Gameshope Loch, Peeblesshire, Scotland, UK (latitude 55.43, longitude –3.38) on 2021-05-26. The specimens were collected and identified by David Bell, David Long and Liz Kungu (Royal Botanic Garden Edinburgh), and preserved by flash freezing in liquid nitrogen. One gametophyte (specimen ID EDTOL01660, ToLID cbStrStra4) was used for Hi-Fi DNA sequencing and another (specimen ID EDTOL01657, ToLID cbStrStra1) was used for Hi-C sequencing. The herbarium specimen associated with the sequenced plant is kept at the Royal Botanic Garden Edinburgh (E) https://data.rbge.org.uk/herb/E01152094.

The initial species identification was verified by an additional DNA barcoding process following the framework developed by Twyford *et al*. (2024). Part of the plant specimen was preserved in silica gel desiccant ( Chase & Hills, 1991). DNA was extracted from the dried specimen, then PCR was used to amplify standard barcode regions. The resulting amplicons were sequenced and compared to public sequence databases including GenBank and the Barcode of Life Database (BOLD). The barcode sequences for this specimen are available on BOLD ( Ratnasingham & Hebert, 2007). Following whole genome sequence generation, DNA barcodes were also used alongside the initial barcoding data for sample tracking through the genome production pipeline at the Wellcome Sanger Institute ( Twyford *et al.*, 2024). The standard operating procedures for the Darwin Tree of Life barcoding have been deposited on protocols.io ( Beasley *et al.*, 2023).

The genome size was estimated by flow cytometry using the fluorochrome propidium iodide and following the ‘one-step’ method as outlined in Pellicer *et al.* (2021). For this species, CyStain™ PI OxProtect Staining Buffer (cat. No. 05-5027; Sysmex UK Ltd.) was used for isolation of nuclei ( Loureiro *et al.*, 2007), and the internal calibration standard was *Oryza sativa* ‘IR36’ with an assumed 1C-value of 493.89 Mb ( Obermayer *et al.*, 2002).

### Nucleic acid extraction

The workflow for high molecular weight (HMW) DNA extraction at the WSI Tree of Life Core Laboratory includes a sequence of procedures: sample preparation and homogenisation, DNA extraction, fragmentation and purification. Detailed protocols are available on protocols.io ( Denton *et al.*, 2023b). The cbStrStra4 sample was weighed and dissected on dry ice ( Jay *et al.*, 2023). Tissue from a shoot of cbStrStra4 was homogenised using a PowerMasher II tissue disruptor ( Denton *et al.*, 2023a). HMW DNA was extracted using the Automated Plant MagAttract v4 protocol ( Jackson & Howard, 2023). HMW DNA was sheared into an average fragment size of 12–20 kb in a Megaruptor 3 system ( Bates *et al.*, 2023). Sheared DNA was purified by solid-phase reversible immobilisation, using AMPure PB beads to eliminate shorter fragments and concentrate the DNA ( Oatley *et al.*, 2023). The concentration of the sheared and purified DNA was assessed using a Nanodrop spectrophotometer, Qubit Fluorometer and Qubit dsDNA High Sensitivity Assay kit. Fragment size distribution was evaluated by running the sample on the FemtoPulse system.

### Hi-C preparation

Hi-C data were generated from shoot tissue of the cbStrStra1 sample at the WSI Scientific Operations core, using the Arima-HiC v2 kit. Tissue was finely ground using cryoPREP, and then subjected to nuclei isolation using a modified protocol of the Qiagen QProteome Kit. After isolation, the nuclei were fixed, and the DNA crosslinked using a 37% formaldehyde solution. The crosslinked DNA was then digested using the restriction enzyme master mix. The 5’-overhangs were then filled in and labelled with biotinylated nucleotides and proximally ligated. An overnight incubation was carried out for enzymes to digest remaining proteins and for crosslinks to reverse. A clean up was performed with SPRIselect beads prior to library preparation. DNA concentration was quantified using the Qubit Fluorometer v2.0 and Qubit HS Assay Kit according to the manufacturer’s instructions.

### Sequencing

Pacific Biosciences HiFi circular consensus DNA sequencing libraries were constructed according to the manufacturers’ instructions. DNA sequencing was performed by the Scientific Operations core at the WSI on a Pacific Biosciences Sequel IIe instrument. Hi-C data were also generated from shoot tissue of cbStrStra1 using the Arima-HiC v2 kit. The Hi-C sequencing was performed using paired-end sequencing with a read length of 150 bp on the Illumina NovaSeq 6000 instrument.

### Library preparation and sequencing

Library preparation and sequencing were performed at the WSI Scientific Operations core. Libraries were prepared using the PacBio Express Template Preparation Kit v2.0 (Pacific Biosciences, California, USA) as per the manufacturer’s instructions. The kit includes the reagents required for removal of single-strand overhangs, DNA damage repair, end repair/A-tailing, adapter ligation, and nuclease treatment. Library preparation also included a library purification step using AMPure PB beads (Pacific Biosciences, California, USA) and size selection step to remove templates shorter than 3 kb using AMPure PB modified SPRI. DNA concentration was quantified using the Qubit Fluorometer v2.0 and Qubit HS Assay Kit and the final library fragment size analysis was carried out using the Agilent Femto Pulse Automated Pulsed Field CE Instrument and gDNA 165 kb gDNA and 55 kb BAC analysis kit. Samples were sequenced using the Sequel IIe system (Pacific Biosciences, California, USA). The concentration of the library loaded onto the Sequel IIe was in the range of 40–135 pM. The SMRT link software, a PacBio web-based end-to-end workflow manager, was used to set-up and monitor the run, as well as perform primary and secondary analysis of the data upon completion.

For Hi-C library preparation, DNA was fragmented to a size of 400 to 600 bp using a Covaris E220 sonicator. The DNA was then enriched, barcoded, and amplified using the NEBNext Ultra II DNA Library Prep Kit, following manufacturers’ instructions. The Hi-C sequencing was performed using paired-end sequencing with a read length of 150 bp on an Illumina NovaSeq 6000.

### Genome assembly, curation and evaluation

***Assembly***

HiFi reads were assembled using the ‘sanger-tol/genomeassembly’ pipeline ( Krasheninnikova *et al.*, 2024). Original assembly of HiFi reads is performed using the Hifiasm ( Cheng *et al.*, 2021) with the --primary option. Haplotypic duplications were identified and removed with purge_dups ( Guan *et al.*, 2020). Hi-C reads are further mapped with bwamem2 ( Vasimuddin *et al.*, 2019) to the primary contigs, which are further scaffolded using the provided Hi-C data ( Rao *et al.*, 2014) in YaHS ( Zhou *et al.*, 2023) using the --break option. Scaffolded assemblies are evaluated using Gfastats ( Formenti *et al.*, 2022), BUSCO ( Manni *et al.*, 2021) and MERQURY.FK ( Rhie *et al.*, 2020).

The organelle genomes were assembled using MitoHiFi ( Uliano-Silva *et al.*, 2023) and OATK ( Zhou, 2023).

***Curation***

The assembly was checked for contamination and corrected using the TreeVal pipeline ( Pointon *et al.*, 2023). Manual curation was performed using JBrowse2 ( Diesh *et al.*, 2023), HiGlass ( Kerpedjiev *et al.*, 2018) and PretextView ( Harry, 2022). The workflow and documentation for rapid curation are provided at https://gitlab.com/wtsi-grit/rapid-curation.

***Evaluation of final assembly***

The final assembly was post-processed and evaluated with the three Nextflow ( Di Tommaso *et al.*, 2017) DSL2 pipelines “sanger-tol/readmapping” ( Surana *et al.*, 2023a), “sanger-tol/genomenote” ( Surana *et al.*, 2023b), and “sanger-tol/blobtoolkit” ( Muffato *et al.*, 2024). The pipeline sanger-tol/readmapping aligns the Hi-C reads with bwa-mem2 ( Vasimuddin *et al.*, 2019) and combines the alignment files with SAMtools ( Danecek *et al.*, 2021). The sanger-tol/genomenote pipeline transforms the Hi-C alignments into a contact map with BEDTools ( Quinlan & Hall, 2010) and the Cooler tool suite ( Abdennur & Mirny, 2020), which is then visualised with HiGlass ( Kerpedjiev *et al.*, 2018). It also computes *k*-mer completeness and QV consensus quality values with FastK and MERQURY.FK, and a completeness assessment with BUSCO ( Manni *et al.*, 2021).

The sanger-tol/blobtoolkit pipeline is a Nextflow port of the previous Snakemake Blobtoolkit pipeline ( Challis *et al.*, 2020). It aligns the PacBio reads with SAMtools and minimap2 ( Li, 2018) and generates coverage tracks for regions of fixed size. In parallel, it queries the GoaT database ( Challis *et al.*, 2023) to identify all matching BUSCO lineages to run BUSCO ( Manni *et al.*, 2021). For the three domain-level BUSCO lineage, the pipeline aligns the BUSCO genes to the Uniprot Reference Proteomes database ( Bateman *et al.*, 2023) with DIAMOND ( Buchfink *et al.*, 2021) blastp. The genome is also split into chunks according to the density of the BUSCO genes from the closest taxonomically lineage, and each chunk is aligned to the Uniprot Reference Proteomes database with DIAMOND blastx. Genome sequences that have no hit are then chunked with seqtk and aligned to the NT database with blastn ( Altschul *et al.*, 1990). All those outputs are combined with the blobtools suite into a blobdir for visualisation.

The genome assembly and evaluation pipelines were developed using the nf-core tooling ( Ewels *et al.*, 2020), use MultiQC ( Ewels *et al.*, 2016), and make extensive use of the Conda package manager, the Bioconda initiative ( Grüning *et al.*, 2018), the Biocontainers infrastructure ( da Veiga Leprevost *et al.*, 2017), and the Docker ( Merkel, 2014) and Singularity ( Kurtzer *et al.*, 2017) containerisation solutions.

Table 4 contains a list of relevant software tool versions and sources.

**Table 4.** Software tools: versions and sources.

Software tool	Version	Source
BEDTools	2.30.0	https://github.com/arq5x/bedtools2
Blast	2.14.0	ftp://ftp.ncbi.nlm.nih.gov/blast/executables/blast+/
BlobToolKit	4.3.7	https://github.com/blobtoolkit/blobtoolkit
BUSCO	5.4.3 and 5.5.0	https://gitlab.com/ezlab/busco
bwa-mem2	2.2.1	https://github.com/bwa-mem2/bwa-mem2
Cooler	0.8.11	https://github.com/open2c/cooler
DIAMOND	2.1.8	https://github.com/bbuchfink/diamond
fasta_windows	0.2.4	https://github.com/tolkit/fasta_windows
FastK	427104ea91c78c3b8b8b49f1a7d6bbeaa869ba1c	https://github.com/thegenemyers/FASTK
Gfastats	1.3.6	https://github.com/vgl-hub/gfastats
GoaT CLI	0.2.5	https://github.com/genomehubs/goat-cli
Hifiasm	0.19.5-r587	https://github.com/chhylp123/hifiasm
HiGlass	44086069ee7d4d3f6f3f0012569789ec138f42b84aa4 4357826c0b6753eb28de	https://github.com/higlass/higlass
Merqury.FK	d00d98157618f4e8d1a9190026b19b471055b22e	https://github.com/thegenemyers/MERQURY.FK
MultiQC	1.14, 1.17, and 1.18	https://github.com/MultiQC/MultiQC
NCBI Datasets	15.12.0	https://github.com/ncbi/datasets
Nextflow	23.04.0-5857	https://github.com/nextflow-io/nextflow
PretextView	0.2	https://github.com/sanger-tol/PretextView
OATK	0.9	https://github.com/c-zhou/oatk
purge_dups	1.2.3	https://github.com/dfguan/purge_dups
samtools	1.16.1, 1.17, and 1.18	https://github.com/samtools/samtools
sanger-tol/ genomeassembly	0.10.0	https://github.com/sanger-tol/genomeassembly
sanger-tol/genomenote	1.1.1	https://github.com/sanger-tol/genomenote
sanger-tol/readmapping	1.2.1	https://github.com/sanger-tol/readmapping
Seqtk	1.3	https://github.com/lh3/seqtk
Singularity	3.9.0	https://github.com/sylabs/singularity
TreeVal	1.0.0	https://github.com/sanger-tol/treeval
YaHS	1.2a.2	https://github.com/c-zhou/yahs

### Wellcome Sanger Institute – Legal and Governance

The materials that have contributed to this genome note have been supplied by a Darwin Tree of Life Partner. The submission of materials by a Darwin Tree of Life Partner is subject to the **‘Darwin Tree of Life Project Sampling Code of Practice’**, which can be found in full on the Darwin Tree of Life website here. By agreeing with and signing up to the Sampling Code of Practice, the Darwin Tree of Life Partner agrees they will meet the legal and ethical requirements and standards set out within this document in respect of all samples acquired for, and supplied to, the Darwin Tree of Life Project. 

Further, the Wellcome Sanger Institute employs a process whereby due diligence is carried out proportionate to the nature of the materials themselves, and the circumstances under which they have been/are to be collected and provided for use. The purpose of this is to address and mitigate any potential legal and/or ethical implications of receipt and use of the materials as part of the research project, and to ensure that in doing so we align with best practice wherever possible. The overarching areas of consideration are:

•   Ethical review of provenance and sourcing of the material

•   Legality of collection, transfer and use (national and international)

Each transfer of samples is further undertaken according to a Research Collaboration Agreement or Material Transfer Agreement entered into by the Darwin Tree of Life Partner, Genome Research Limited (operating as the Wellcome Sanger Institute), and in some circumstances other Darwin Tree of Life collaborators.

## Data Availability

European Nucleotide Archive: *Straminergon stramineum* (straw spear-moss). Accession number PRJEB70978; https://identifiers.org/ena.embl/PRJEB70978. The genome sequence is released openly for reuse. The *Straminergon stramineum* genome sequencing initiative is part of the Darwin Tree of Life (DToL) project. All raw sequence data and the assembly have been deposited in INSDC databases. The genome will be annotated using available RNA-Seq data and presented through the Ensembl pipeline at the European Bioinformatics Institute. Raw data and assembly accession identifiers are reported in Table 1.

## Author information

Members of the Royal Botanic Garden Edinburgh Genome Acquisition Lab are listed here: https://doi.org/10.5281/zenodo.4786682.

Members of the Plant Genome Sizing collective are listed here: https://doi.org/10.5281/zenodo.7994306.

Members of the Darwin Tree of Life Barcoding collective are listed here: https://doi.org/10.5281/zenodo.4893703.

Members of the Wellcome Sanger Institute Tree of Life Management, Samples and Laboratory team are listed here: https://doi.org/10.5281/zenodo.10066175.

Members of Wellcome Sanger Institute Scientific Operations: Sequencing Operations are listed here: https://doi.org/10.5281/zenodo.10043364.

Members of the Wellcome Sanger Institute Tree of Life Core Informatics team are listed here: https://doi.org/10.5281/zenodo.10066637.

Members of the Tree of Life Core Informatics collective are listed here: https://doi.org/10.5281/zenodo.5013541.

Members of the Darwin Tree of Life Consortium are listed here: https://doi.org/10.5281/zenodo.4783558.

## References

[ref-1] Abdennur N Mirny LA : Cooler: scalable storage for Hi-C data and other genomically labeled arrays. *Bioinformatics.* 2020;36(1):311–316. 10.1093/bioinformatics/btz540 31290943 PMC8205516

[ref-2] Altschul SF Gish W Miller W : Basic local alignment search tool. *J Mol Biol.* 1990;215(3):403–410. 10.1016/S0022-2836(05)80360-2 2231712

[ref-3] Bateman A Martin MJ Orchard S : UniProt: the universal protein knowledgebase in 2023. *Nucleic Acids Res.* 2023;51(D1):D523–D531. 10.1093/nar/gkac1052 36408920 PMC9825514

[ref-4] Bates A Clayton-Lucey I Howard C : Sanger Tree of Life HMW DNA fragmentation: diagenode Megaruptor ^®^3 for LI PacBio. *protocols.io.* 2023. 10.17504/protocols.io.81wgbxzq3lpk/v1

[ref-5] Beasley J Uhl R Forrest LL : DNA barcoding SOPs for the Darwin Tree of Life project. *protocols.io.* 2023; [Accessed 25 June 2024]. 10.17504/protocols.io.261ged91jv47/v1

[ref-6] Blockeel TL Bosanquet SDS Hill MO : Atlas of British and Irish bryophytes.Newbury: Pisces Publications,2014. Reference Source

[ref-7] Buchfink B Reuter K Drost HG : Sensitive protein alignments at Tree-of-Life scale using DIAMOND. *Nat Methods.* 2021;18(4):366–368. 10.1038/s41592-021-01101-x 33828273 PMC8026399

[ref-8] Challis R Kumar S Sotero-Caio C : Genomes on a Tree (GoaT): a versatile, scalable search engine for genomic and sequencing project metadata across the eukaryotic Tree of Life [version 1; peer review: 2 approved]. *Wellcome Open Res.* 2023;8:24. 10.12688/wellcomeopenres.18658.1 36864925 PMC9971660

[ref-9] Challis R Richards E Rajan J : BlobToolKit – interactive quality assessment of genome assemblies. *G3 (Bethesda).* 2020;10(4):1361–1374. 10.1534/g3.119.400908 32071071 PMC7144090

[ref-10] Chase MW Hills HH : Silica gel: an ideal material for field preservation of leaf samples for DNA studies. *Taxon.* 1991;40(2):215–220. 10.2307/1222975

[ref-11] Cheng H Concepcion GT Feng X : Haplotype-resolved *de novo* assembly using phased assembly graphs with hifiasm. *Nat Methods.* 2021;18(2):170–175. 10.1038/s41592-020-01056-5 33526886 PMC7961889

[ref-12] da Veiga Leprevost F Grüning BA Alves Aflitos S : BioContainers: an open-source and community-driven framework for software standardization. *Bioinformatics.* 2017;33(16):2580–2582. 10.1093/bioinformatics/btx192 28379341 PMC5870671

[ref-13] Danecek P Bonfield JK Liddle J : Twelve years of SAMtools and BCFtools. *GigaScience.* 2021;10(2): giab008. 10.1093/gigascience/giab008 33590861 PMC7931819

[ref-14] Denton A Oatley G Cornwell C : Sanger Tree of Life sample homogenisation: PowerMash. *protocols.io.* 2023a. 10.17504/protocols.io.5qpvo3r19v4o/v1

[ref-15] Denton A Yatsenko H Jay J : Sanger Tree of Life wet laboratory protocol collection V.1. *protocols.io.* 2023b. 10.17504/protocols.io.8epv5xxy6g1b/v1

[ref-16] Di Tommaso P Chatzou M Floden EW : Nextflow enables reproducible computational workflows. *Nat Biotechnol.* 2017;35(4):316–319. 10.1038/nbt.3820 28398311

[ref-17] Diesh C Stevens GJ Xie P : JBrowse 2: a modular genome browser with views of synteny and structural variation. *Genome Biol.* 2023;24(1): 74. 10.1186/s13059-023-02914-z 37069644 PMC10108523

[ref-19] Ewels P Magnusson M Lundin S : MultiQC: summarize analysis results for multiple tools and samples in a single report. *Bioinformatics.* 2016;32(19):3047–3048. 10.1093/bioinformatics/btw354 27312411 PMC5039924

[ref-18] Ewels PA Peltzer A Fillinger S : The nf-core framework for community-curated bioinformatics pipelines. *Nat Biotechnol.* 2020;38(3):276–278. 10.1038/s41587-020-0439-x 32055031

[ref-20] Formenti G Abueg L Brajuka A : Gfastats: conversion, evaluation and manipulation of genome sequences using assembly graphs. *Bioinformatics.* 2022;38(17):4214–4216. 10.1093/bioinformatics/btac460 35799367 PMC9438950

[ref-21] Fritsch R : Index to bryophyte chromosome counts. 1991;40. Reference Source

[ref-22] Grüning B Dale R Sjödin A : Bioconda: sustainable and comprehensive software distribution for the life sciences. *Nat Methods.* 2018;15(7):475–476. 10.1038/s41592-018-0046-7 29967506 PMC11070151

[ref-23] Guan D McCarthy SA Wood J : Identifying and removing haplotypic duplication in primary genome assemblies. *Bioinformatics.* 2020;36(9):2896–2898. 10.1093/bioinformatics/btaa025 31971576 PMC7203741

[ref-24] Harry E : PretextView (Paired REad TEXTure Viewer): a desktop application for viewing pretext contact maps. 2022. Reference Source

[ref-25] Hedenäs L : A generic revision of the *Warnstorfia-Calliergon* group. *J Bryol.* 1993;17(3):447–479. 10.1179/jbr.1993.17.3.447

[ref-26] Jackson B Howard C : Sanger Tree of Life HMW DNA extraction: automated plant MagAttract v.4. *protocols.io.* 2023. 10.17504/protocols.io.8epv5xrd5g1b/v1

[ref-27] Jay J Yatsenko H Narváez-Gómez JP : Sanger Tree of Life sample preparation: triage and dissection. *protocols.io.* 2023. 10.17504/protocols.io.x54v9prmqg3e/v1

[ref-28] Kerpedjiev P Abdennur N Lekschas F : HiGlass: web-based visual exploration and analysis of genome interaction maps. *Genome Biol.* 2018;19(1): 125. 10.1186/s13059-018-1486-1 30143029 PMC6109259

[ref-29] Krasheninnikova K Qi G Muffato M : sanger-tol/genomeassembly: v0.10.0 - Hideous Zippleback. *Zenodo.* 2024. 10.5281/zenodo.10990898

[ref-30] Kurtzer GM Sochat V Bauer MW : Singularity: scientific containers for mobility of compute. *PLoS One.* 2017;12(5): e0177459. 10.1371/journal.pone.0177459 28494014 PMC5426675

[ref-31] Li H : Minimap2: pairwise alignment for nucleotide sequences. *Bioinformatics.* 2018;34(18):3094–3100. 10.1093/bioinformatics/bty191 29750242 PMC6137996

[ref-32] Loureiro J Rodriguez E Dolezel J : Two new nuclear isolation buffers for plant DNA flow cytometry: a test with 37 species. *Ann Bot.* 2007;100(4):875–888. 10.1093/aob/mcm152 17684025 PMC2749623

[ref-33] Manni M Berkeley MR Seppey M : BUSCO update: novel and streamlined workflows along with broader and deeper phylogenetic coverage for scoring of eukaryotic, prokaryotic, and viral genomes. *Mol Biol Evol.* 2021;38(10):4647–4654. 10.1093/molbev/msab199 34320186 PMC8476166

[ref-34] Merkel D : Docker: lightweight Linux containers for consistent development and deployment. *Linux J.* 2014;2014(239): 2, [Accessed 2 April 2024]. Reference Source

[ref-35] Muffato M Butt Z Challis R : sanger-tol/blobtoolkit: v0.3.0 – Poliwag. 2024. 10.5281/zenodo.10649272

[ref-36] Oatley G Sampaio F Howard C : Sanger Tree of Life fragmented DNA clean up: automated SPRI. *protocols.io.* 2023. 10.17504/protocols.io.q26g7p1wkgwz/v1

[ref-37] Obermayer R Leitch IJ Hanson L : Nuclear DNA C-values in 30 species double the familial representation in pteridophytes. *Ann Bot.* 2002;90(2):209–217. 10.1093/aob/mcf167 12197518 PMC4240412

[ref-38] Pellicer J Powell RF Leitch IJ : The application of flow cytometry for estimating genome size, ploidy level endopolyploidy, and reproductive modes in plants. *Methods Mol Biol.* In: Besse, P. (ed.) New York, NY: Humana,2021;2222:325–361. 10.1007/978-1-0716-0997-2_17 33301101

[ref-39] Pointon DL Eagles W Sims Y : sanger-tol/treeval v1.0.0 – Ancient Atlantis. 2023. 10.5281/zenodo.10047654

[ref-40] Quinlan AR Hall IM : BEDTools: a flexible suite of utilities for comparing genomic features. *Bioinformatics.* 2010;26(6):841–842. 10.1093/bioinformatics/btq033 20110278 PMC2832824

[ref-41] Rao SSP Huntley MH Durand NC : A 3D map of the human genome at kilobase resolution reveals principles of chromatin looping. *Cell.* 2014;159(7):1665–1680. 10.1016/j.cell.2014.11.021 25497547 PMC5635824

[ref-42] Ratnasingham S Hebert PDN : bold: the barcode of Life data system ( http://www.barcodinglife.org). *Mol Ecol Notes.* 2007;7(3):355–364. 10.1111/j.1471-8286.2007.01678.x 18784790 PMC1890991

[ref-43] Rhie A McCarthy SA Fedrigo O : Towards complete and error-free genome assemblies of all vertebrate species. *Nature.* 2021;592(7856):737–746. 10.1038/s41586-021-03451-0 33911273 PMC8081667

[ref-44] Rhie A Walenz BP Koren S : Merqury: reference-free quality, completeness, and phasing assessment for genome assemblies. *Genome Biol.* 2020;21(1): 245. 10.1186/s13059-020-02134-9 32928274 PMC7488777

[ref-45] Surana P Muffato M Qi G : Sanger-tol/readmapping: sanger-tol/readmapping v1.1.0 - Hebridean Black (1.1.0). *Zenodo.* 2023a. 10.5281/zenodo.7755669

[ref-46] Surana P Muffato M Sadasivan Baby C : sanger-tol/genomenote (v1.0.dev). *Zenodo.* 2023b. 10.5281/zenodo.6785935

[ref-47] Twyford AD Beasley J Barnes I : A DNA barcoding framework for taxonomic verification in the darwin Tree of Life project [version 1; peer review: 2 approved]. *Wellcome Open Res.* 2024;9:339. 10.12688/wellcomeopenres.21143.1 39386966 PMC11462125

[ref-48] Uliano-Silva M Ferreira JGRN Krasheninnikova K : MitoHiFi: a python pipeline for mitochondrial genome assembly from PacBio high fidelity reads. *BMC Bioinformatics.* 2023;24(1): 288. 10.1186/s12859-023-05385-y 37464285 PMC10354987

[ref-49] Vasimuddin M Misra S Li H : Efficient architecture-aware acceleration of BWA-MEM for multicore systems.In: *2019 IEEE International Parallel and Distributed Processing Symposium (IPDPS).*IEEE,2019;314–324. 10.1109/IPDPS.2019.00041

[ref-50] Voglmayr H : Nuclear DNA amounts in mosses ( *Musci*). *Ann Bot.* 2000;85(4):531–546. 10.1006/anbo.1999.1103

[ref-51] Zhou C : c-zhou/oatk: Oatk-0.1. 2023. 10.5281/zenodo.7631375

[ref-52] Zhou C McCarthy SA Durbin R : YaHS: yet another Hi-C scaffolding tool. *Bioinformatics.* 2023;39(1): btac808. 10.1093/bioinformatics/btac808 36525368 PMC9848053

