# Supplementary material for: The genome sequence of the straw spear-moss, Straminergon stramineum (Dicks. ex Brid.) Hedenas, 1993
Source: Wellcome Open Res. 2024 Dec 3;9:712. [Version 1] doi: 10.12688/wellcomeopenres.23432.1 (PMC11992514; doi:10.12688/wellcomeopenres.23432.1)
Supplement: Peer review history

# Reviewer report: Reviewer response for version 1

**DOI:** 10.21956/wellcomeopenres.25842.r121340

**Published:** 2025-04-11

**Authors:** Cymon J Cox

**Affiliations:** 1 Centro de Ciencias do Mar, Faro, Portugal

The authors present a Data Note describing the sequencing and assembly features of the genome of the pleurocarpous moss, *Straminergon stramineum*. The description of the methods and assembly characteristics are detailed, thorough, and appropriately referenced. The resulting genomic data appear to be of high quality, but, as intended with such notes, there is no discussion or interpretation of the presented data to comment on. Commendably, the Darwin Tree of Life project publishes data with an exemplar degree of openness and attention to the data being FAIR and potentially interoperable within and between data domains.

Are sufficient details of methods and materials provided to allow replication by others?

Yes

Is the rationale for creating the dataset(s) clearly described?

Yes

Are the datasets clearly presented in a useable and accessible format?

Yes

Are the protocols appropriate and is the work technically sound?

Yes

Reviewer Expertise:

Plant Systematic, bioinformatics, open science advocacy.

I confirm that I have read this submission and believe that I have an appropriate level of expertise to confirm that it is of an acceptable scientific standard.

# Reviewer report: Reviewer response for version 1

**DOI:** 10.21956/wellcomeopenres.25842.r121341

**Published:** 2025-04-09

**Authors:** Facundo Romani

**Affiliations:** 1 University of Cambridge, Cambridge, UK

As other data notes from the Tree of Life Project, the assembly of a new bryophyte genome is very welcome. The quality of the genome assembly is very high with state of the art technologies and methods. The way it is presented can always be a bit better. The dataset include an RNA-seq but that was not included in this work. The annotation of the genome with the RNA-seq would make the data much more accessible for researchers interesting on doing BLAST.

Are sufficient details of methods and materials provided to allow replication by others?

Yes

Is the rationale for creating the dataset(s) clearly described?

Yes

Are the datasets clearly presented in a useable and accessible format?

Partly

Are the protocols appropriate and is the work technically sound?

Yes

Reviewer Expertise:

Bryologists and evolution of development.

I confirm that I have read this submission and believe that I have an appropriate level of expertise to confirm that it is of an acceptable scientific standard.
